# Patients’ perspectives on the quality of care of a new complex psycho-oncological care programme in Germany – external mixed methods evaluation results

**DOI:** 10.1186/s12913-023-09714-y

**Published:** 2023-07-15

**Authors:** Natalia Cecon-Stabel, Sandra Salm, Holger Pfaff, Antje Dresen, Theresia Krieger

**Affiliations:** 1 Faculty of Medicine and University Hospital Cologne, Faculty of Human Sciences, Institute for Medical Sociology, Health Services Research, and Rehabilitation Science (IMVR), University of Cologne, Cologne, Germany; 2 Goethe University Frankfurt, Institute of General Practice, Frankfurt, Germany; 3 Medical Psychology, Neuropsychology and Gender Studies and Center for Neuropsychological Diagnostics and Intervention (CeNDI), Faculty of Medicine and University Hospital Cologne, University of Cologne, Cologne, Germany

**Keywords:** Psycho-oncology, Complex intervention, Quality of care, Patient perspective, Evaluation, Mixed methods

## Abstract

**Background:**

Guideline-compliant provision of psycho-oncological (PO) care is still challenging in Germany. Hence, a new care programme, called integrated cross-sectoral psycho-oncology (isPO), was implemented to improve the integration of needs-oriented PO care. Quality of care (QoC) was externally evaluated from the patient’s perspective. We aim to gain insight into patients’ experiences with isPO and how their assessment affects relevant patient-reported outcomes (anxiety and depression, health status, and work ability).

**Methods:**

An explanatory, sequential mixed-methods design was applied. Patients were surveyed twice during their 1-year care trajectory: after 3 (T1) and 12 (T2) months. Data sets were matched using pseudonyms. Care documentation data, including sociodemographic characteristics and the primary outcome variable (anxiety and depression), were matched. In the survey, patients rated their satisfaction with respective isPO service providers and the programme in general (QoC). Health status (EORTC-QLQ-C30) and work ability (WAS) were measured. Descriptive analyses and t-tests for dependent samples were conducted to assess changes in outcome variables over time. Linear regression analyses were conducted to assess whether care satisfaction predicted outcome variables. Patients who completed their isPO care trajectory were asked to participate in semi-structured telephone interviews to share their experiences. Purposeful sampling was applied. All 23 interviews were audiotaped, transcribed, and analysed via content-structuring method.

**Results:**

Patients reported medium-to-high satisfaction with their isPO care. All patient-related outcomes significantly improved over time and QoC measures predicted those outcomes. Needs orientation (e.g., care intensity or mode of delivery) was perceived as essential for high QoC, and outpatient care with fixed contact persons as highly important for care continuity. Furthermore, patients identified programme optimisation needs, such as period of care or extension of care to relatives.

**Conclusions:**

Patients assessed the isPO programme’s QoC positively. They identified facilitators for QoC and optimisation needs. Therefore, data on QoC can function as an indicator for a programme’s feasibility and maturity within care reality. As patients’ care satisfaction positively influences important patient-related outcomes, it may be routinely considered for quality management. Based on patients’ perspectives, isPO seems to be recommendable for routine psycho-oncological care in Germany, if ongoing programme optimisation within structured quality management is guaranteed.

**Trial registration:**

The study was registered in the German Clinical Trials Register (No. DRKS00015326) on 30.10.2018.

**Supplementary Information:**

The online version contains supplementary material available at 10.1186/s12913-023-09714-y.

## Background

Many cancer patients suffer from distress, fatigue, anxiety, depression or posttraumatic stress [1–3]. Emotional distress is recognised as ‘The 6th Vital Sign’ in cancer care [4] and has led to the implementation of screening instruments and evidence-based psycho-oncological interventions worldwide [5]. Psycho-oncological care includes a multidisciplinary approach, entailing psychological, social, behavioural, and ethical aspects [6, 7]. Although many psycho-oncological interventions have been developed, implementing them into practice still remains a challenge [8]. Hence, research is needed that considers clinical, social and cultural context of cancer, including research on the dissemination and evaluation of interventions in different countries [5].

### Psycho-oncological care in Germany

In Germany, only a fraction of cancer patients receives adequate psycho-oncological (PO) care [9, 10], despite one in two cancer patients experiencing significant distress [11]. Guideline-compliant provision and implementation of PO care is still considered challenging [12]. First, there is currently no legal basis for uniform, area- and cost-covering financing [13, 14]. Furthermore, psycho-oncology is not offered nationwide in Germany; rural areas are especially underserved [15]. Moreover, there is a strong sectoral separation of PO care structures between and within health and social services [7, 16–19]. There is a lack of nationwide expansion of cancer counselling centres regarding psychosocial area coverage, qualified counsellors, and equally secure funding [15, 20]. For these reasons, the national cancer plan [21] calls for cross-sectoral and needs-oriented integration of PO care into oncological care. The new form of care, named ‘integrated cross-sectoral psycho-oncology’ (isPO), aims to follow the national cancer plan’s call by integrating such structures to reduce the described challenges in the future [22].

### The isPO intervention programme and its evaluation

The isPO intervention programme was developed, implemented, and externally evaluated between 2018 and 2022 [22, 23]. At a patient level, the programme aims to reduce anxiety and depression in newly diagnosed cancer patients based on their individual needs within a 1-year care period (stepped-care approach). At the health-system level, the isPO project aims to develop a high-quality PO programme that may be available as an integrated, cross-sectoral form of care for cancer patients for possible adoption into standard nationwide care. For this, multiple programme components (Table 1) were developed that relate to different aspects of care (structural, processual, clinical, and legal): a stepped care concept, including new care pathways; newly established PO care networks and care process organisation; a newly developed information technology-supported care documentation and assistance system called ‘CAPSYS^2020^’, which supports PO service providers with billing, care coordination, and documentation; and isPO-specific quality assurance and improvement structures [22, 23].

**Table 1:** The isPO programme’s components

Programme component	Description
Stepped care concept	The care concepts’ development is based on the effect theory according to Issel [25] and consists of one general and four minor care concepts [22]. Details on the causal, intervention and impact theory underlying the concept(s) are published by Kusch et al. [22]. In general, the intervention theory is based on a stepped care approach [7, 26–28], in which the intervention measures are assigned to patients’ care needs. Based on the Hospital Anxiety and Depression scale [29] and the Psychosocial Risk questionnaire [30] patients are allocated to a specific care level (Fig. 1). All patients are supported by isPO-specific case management services and are offered a conversation with a so-called isPO onco-guide (trained former cancer survivor) [22]. Dependent on the individual care needs, patients may additionally receive psychosocial or psycho-oncological-psychotherapeutic care [22]. Patients with complex care needs, may receive both, psychosocial and psycho-oncological-psychotherapeutic care [22]. Care services within the isPO programme are limited to one year [22].
Care pathways	The isPO programme contains general care pathways (Fig. 1) and detailed minor care pathways [22]. Each existing care pathway includes an algorithm with specific execution and selection recommendations which is integrated into the information technology (IT) – supported documentation and assistance system ‘CAPSYS’. Within CAPSYS, there are specific care documents filed for each care pathway, for instance: the isPO care manual, instructions, or evaluations of the deployed psychometric instruments. The structure of the care pathways mainly aims to ensure contractually appropriate care delivery and quality assurance.
Psycho-oncological care networks	The isPO programme aims to be integrated into bio-medical care and across different phases of cancer treatment (from acute therapy to aftercare) [22, 23]. For this, new psycho-oncological care networks were established. Each network consists of one cancer centre hospital that cooperates with at least one outpatient oncological practice. Hence, cancer patients may be referred to the isPO programme regardless in which setting their biomedical treatment takes place. The establishment of the care networks is contractually regulated.
Care process organisation	Psycho-oncological care that is provided within the isPO programme is contractually specified with German health insurance agencies [22]. This care contract refers to isPO’s core clinical services and clinical processes. Further, it specifies core formal and administrative services. Core services and processes were operationalised in the form of selection and execution recommendations that map the care concept [22]. Local tailoring of the recommendations is possible to achieve a good fit for the implementation setting [22].
Information technology (IT)-supported documentation and assistance system CAPSYS	CAPSYS aims to meet national care requirements (SGB V) and internal needs of care documentation, care and quality management, billing and data protection [22]. It consists of two parts that are interlinked: CAPSYS-docu and CAPSYS-assist. CAPSYS-docu may be used for capturing patient care data and care service delivery. CAPSYS-assist was developed to support the planning, guidance and examination of patient care [22]. Other components of the isPO programme, e.g. care pathways or care process organisation, are integrated in CAPSYS.
Quality assurance and improvement	Every quarter, internal quality circles are held in the psycho-oncological care networks to ensure quality of care [22]. Further, external quality workshops are conducted with the consortium partners of the isPO project (e.g. programme designer and implementation supporters) and representatives from the psycho-oncological care networks (usually the network coordinators and head psycho-oncologists) [22]. In addition, quality indicators were defined based on the operational, clinical and formal-administrative recommendations of the care pathways [22]. Data to assess the indicators stem from the care data in CAPSYS, which then allows the creation of quality reports and benchmarking [22].

According to definitions on complex interventions [24], isPO can be considered a complex care programme. The two components ‘care concept’ and ‘care pathways’ represent the clinical care aspects, whereas the other components represent formal administrative aspects of PO care that aim to meet legal requirements for care in the German healthcare system [22].

**Fig. 1:**
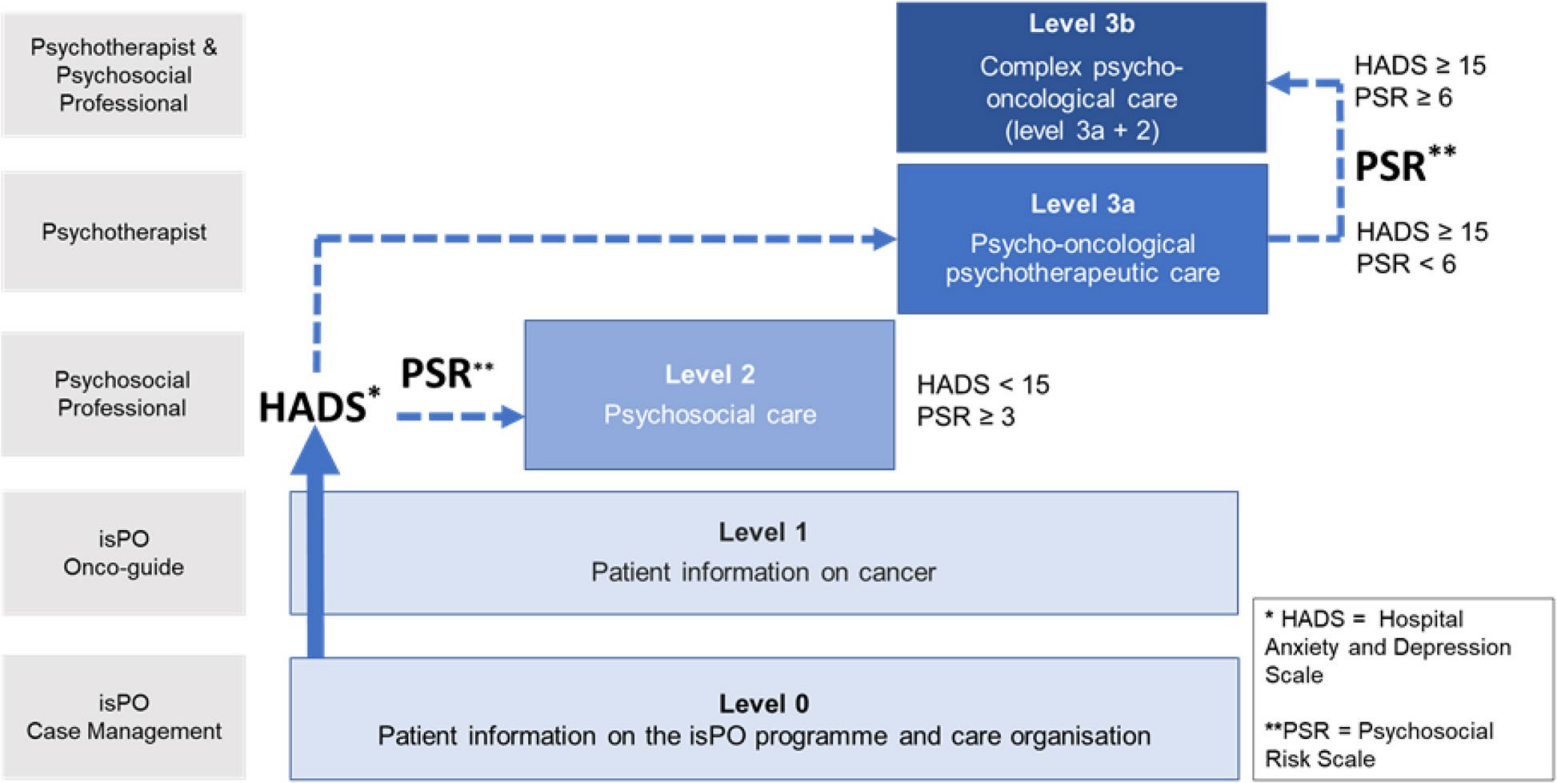
The isPO programme’s stepped care concept. Adapted from Kusch et al. [22] and Salm and Cecon et al. [31]

In 2019, programme implementation began in four newly established PO care networks in North Rhine-Westphalia. They each consisted of a cooperation between at least one certified oncological cancer centre hospital and local oncological practices (see Table 1). Physicians referred patients who received their cancer diagnosis in the care networks to the isPO programme. During programme enrolment, patients’ degree of distress in terms of anxiety, depression, and psychosocial risk factors were screened to allocate them to a care level that was designed to meet their individual needs [22, 23]. Dependent on the assigned care level, various professions were involved in isPO care provision: licensed psychotherapists, psychosocial professionals, case managers, and specially trained cancer survivors called ‘isPO onco-guides’. Figure 1 illustrates core isPO care pathways of the care concept.

The isPO programme was externally evaluated [23]. The evaluation process is based on the Medical Research Council framework for the analysis and assessment of complex interventions [32]. A comprehensive study was interlinked with the programme to evaluate its effectiveness and quality of care. Quality of care is defined as the extent to which care is provided to patients in a matter that achieves the desired health-related outcomes and is consistent with current knowledge [33]. In this regard, quality of care may be divided into structural, processual, and outcome quality [34]. Quality of care can be assessed with a mixed-methods design [23, 35–37].

### Objective

In this article, we report on the assessment of the quality of care of the isPO programme from patients’ perspectives, which is part of the external evaluation of isPO. We aimed to gain deeper insight into patients’ individual isPO programme experiences, how they assessed the programme, and whether assessment affected relevant patient-reported outcomes.

## Methods

**Fig. 2:**
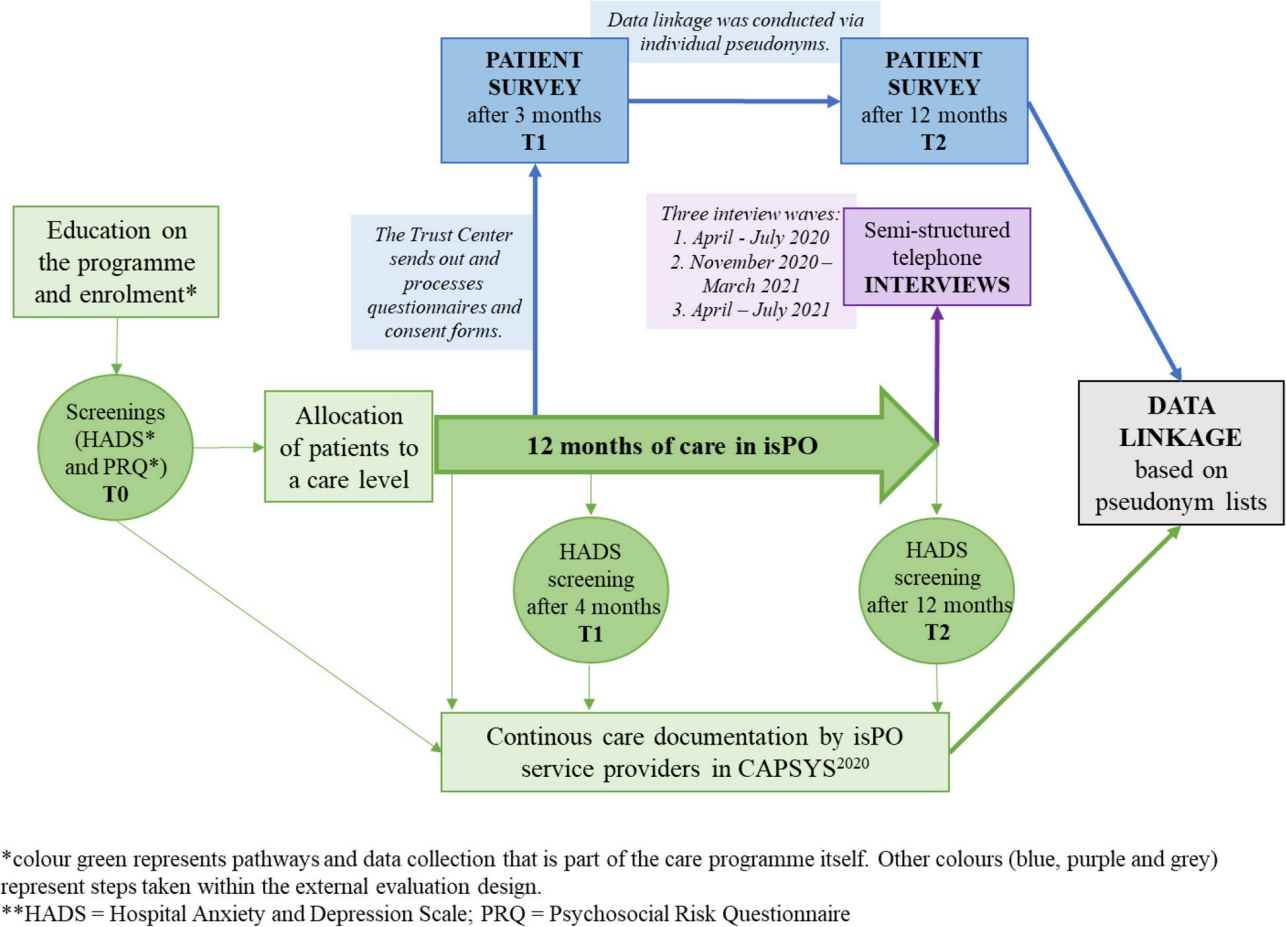
Mixed-methods design to assess patients’ perspectives on the integrated cross-sectoral psycho-oncology (isPO) programme

An explanatory sequential mixed methods design [38] with qualitative and quantitative methods was applied to assess patients’ perspectives on quality of care in isPO (Fig. 2).

During enrolment, patients could consent: (1) to participate in the isPO programme, (2) to share their data (e.g. documentation data or statutory health data) with the interlinked evaluation study as well as (3) to be contacted for evaluation surveys or interviews. Patients who enrolled in the programme, were allocated to a care level based on their individual needs, which were assessed with screening instruments. Screening results and any care documentation were saved in CAPSYS^2020^ by the isPO service providers. Using multiple pseudonyms, CAPSYS^2020^ data were linked with primary quantitative data collected via external evaluation whilst conforming to German data protection laws [23]. Primary data collection included a patient survey with two measurement times. Furthermore, a sample of patients who finished their 1-year care in the isPO programme were interviewed about their individual care experiences. To gain differentiated insight in the care experiences, we aimed to consider the different periods of implementation, e.g. first year of implementation, after programme optimisations have been made or after supposedly a routinisation has occurred in care delivery. Additionally, the coronavirus disease 2019 (COVID-19) pandemic occurred in the beginning of the second year of implementation, which we also considered in the evaluation. For this, three patient interview waves were conducted. The study procedure was approved by the ethics committee of the Medical Faculty of the University of Cologne.

### Patient survey

isPO patients who enrolled and consented to being contacted for a survey or an interview were contacted by the isPO Trust Center. They were questioned twice during their 12 month-long care: 3 months into their care and at the end of their care (12 months). The isPO Trust Center contacted 1599 enrolled patients with a consent form and questionnaire by post 3 months after enrolment. Patients who wished to participate could return their completed questionnaire and consent form in two pre-stamped envelopes. Dillman’s Total Design Method [39] was applied to achieve the highest possible response rate. For this, patients were contacted twice after initial send-out of the questionnaire. This means, that after 2 weeks, patients received a postcard with a survey reminder, and 3 weeks after the postcard, they received a reminder with a new questionnaire and consent form. The Trust Center allocated a survey pseudonym (SP) to each patient so that patients who participated in the first survey could be contacted for the second one. The survey data were imported into SPSS [40] for data analysis.

### Measurements

To measure quality of care, we considered variables regarding patients’ satisfaction with the different isPO service providers (case management, isPO onco-guides, psychosocial professionals, and psychotherapists) and general assessment of patient care in isPO (subjective effectiveness, satisfaction and needs orientation, frequency and duration of appointments). Furthermore, patient-reported outcomes (global health status, work ability, anxiety and depression) and sociodemographic variables were reported. Table 2 provides an overview of the used measurements. For sample characteristics, the following variables were linked from the CAPSYS^2020^ data set: age, sex, ISCED index, care network (pseudonymised via number 1 to 4), and anxiety and depression.

**Table 2:** Overview and specifics on the scales and items used to assess quality of care and outcomes from the isPO patients’ perspective

Variable & time point of measurement	Scale and item specifics	Example item
Satisfaction with case management T1^a^	health literacy-sensitivity of communication scale (HL-COM) [41] 8 items^b^ Four point Likert scale from 1 ‘I totally disagree’ to 4’I fully agree’	I was encouraged to ask questions if I did not understand something
Satisfaction with the isPO onco-guide T1	Two items^c^ from the Consultation and Relational Empathy Scale (CARE) [42] were adapted and used Five additional self-developed items were used to evaluate whether the… - information provided by the onco-guide was helpful - onco-guide answered questions in a satisfactory manner - onco-guide had enough time for the patient - patient felt connected to the onco-guide - and how satisfied the patient was overall Four point Likert scale from 1 ‘I totally disagree’ to 4’I fully agree’	The isPO onco-guide behaved in a way that made me feel comfortable
Satisfaction with the psychosocial professional T2	Six self-developed items were used to evaluate, whether… - patients received helpful information during their consultations - information was explained in an understandable way - the psychosocial professional answered all the patient’s questions - the consultations were too short - the patient’s personal circumstances and environment were taken into account - patients were advised in a way that they were able to put advice and support into practice Four point Likert scale from 1 ‘I totally disagree’ to 4’I fully agree’	The psychosocial professional explained all the information to me in an understandable way
Satisfaction with the psychotherapist (therapeutic alliance) T2	12-item short German version of the Working Alliance Inventory (WAI-SR) [43], which is based on the English WAI-SR [44].^d^ The inventory measures the dimensions of therapeutic alliance, as described by Bordin [45], including: Goal, Task, and Bond Patients indicated how often an item applied on a five-point Likert scale	We agree on what is important for me to work on
Subjective effectiveness T2	Four self-developed items Four-point Likert scale from 1 ‘I totally disagree’ to 4’I fully agree’	The care in isPO makes me feel better
Satisfaction and orientation to needs T2	Four self-developed items Four-point Likert scale from 1 ‘I totally disagree’ to 4’I fully agree’	The care in isPO supported me according to my needs
Frequency of appointments T2	One self-developed item Three response options: ‘too rare’, ‘suitable’, ‘too often’	The frequency of appointments was…
Duration of appointments T2	One self-developed item Three response options: ‘too short’, ‘suitable’, ‘too long’	The duration of appointments was…
Global health status T1 & T2	Global health status subscale consisting of two items of the German version of the EORTC-QLQ-C-30 [46] Response options from 1 ‘very bad’ to 7’excellent’ Transformed scores can range from 0 to 100. Higher scores represent a better level of functioning or less intense level of symptoms	Overall, how would you rate your physical status during the last week?
Work ability T1 & T2	Work Ability score (WAS), which consists of one item [47, 48] Patients assessed their current work ability compared to their lifetime best: poor (0–5 points), moderate (6–7 points), good (8–9 points), or excellent (10) from 0 ‘completely unfit for work’ to 10 ‘currently best work ability’	If you rate your best ever work ability with 10 points: how many points would you give for your current working ability?
Anxiety and depression T0, T1, T2^e^	German version of the Hospital Anxiety and Depression Scale (HADS) [29] The scale captures the degree of anxious and depressive symptoms in the past week and consists of two subscales, Anxiety and Depression, with seven items each	Worrying thought go through my mind

^a^only measured in the first patient survey, as patients usually had most of the contact in the beginning of their PO care

^b^the item ‘my results were explained comprehensively to me.’ was excluded from the usually 9 item-long scale, as it was not relevant for isPO case management

c‘The onco-guide behaved in a way that made me feel comfortable’ and ‘The onco-guide was interested in me as a person and in my environment’

^d^‘What I am doing in therapy gives me new ways of looking at my problem’ and ‘I believe the way we are working with my problem is correct’ were adapted to ‘What I am doing in therapy gives me new ways of looking at my way of dealing with the disease’ and ‘I believe the way we are working with how I deal with my cancer is correct’

^e^T0 = at programme enrolment, T1 = 4 months into care and T2 = after 12 months (at the end of care). Patients were instructed to fill out the HADS independently of the external evaluation patient survey. HADS value at T0 determined to which care level a patient was allocated

### Statistical analysis

Descriptive data analysis (frequencies, mean, standard deviation, minimum and maximum) was first conducted for patients’ satisfaction with their respective isPO service providers and the scales concerning their care in isPO in general. Next, to determine the differences in global health status, anxiety and depression, and work ability between the survey time points, t-tests for dependent samples were calculated. Finally, linear regression analyses were conducted to assess whether quality of care predicted or was associated with global health status, anxiety, depression, and work ability. Items concerning frequency and duration of appointments were initially categorical; however, the third category ‘too often’ and ‘too long’ was empty or only chosen by one person. Therefore, it was possible to dummy code it to 1’suitable’ and 0 ‘not suitable’.

### Patient interviews

#### Data collection

Due to the different stages of implementation and the COVID-19 pandemic, three interview waves were established. The first interview wave was conducted between April and July 2020 to consider care experiences made in the first year of programme implementation, during which programme optimisations were conducted. The second interview wave was conducted between November 2020 and March 2021 to consider the second year of implementation, which showed more routinisation in care delivery. Lastly, the third interview wave was conducted from April to June 2021 to consider care experiences made under pandemic conditions (COVID-19 pandemic). In all, we conducted 23 telephone interviews, of which 9 were in the first, 10 in the second, and 4 in the third interview wave. Because the pandemic started around March 2020 in Germany, all semi-structured interviews needed to be conducted via telephone.

Purposeful sampling [49] was applied. Patients were recruited from all care networks, with different cancer entities and according to gender, age, and intensity of isPO care (e.g. number of appointments or isPO care stage). Patients were first approached by their care service provider, e.g. psychotherapist, during a face-to-face or telephonic appointment and asked, if they wanted to share their care experiences with the external evaluation team. It was explained to them that the isPO programme is being evaluated on its quality of care and that the interviews are part of this evaluation process. If patients agreed, the isPO Trust Center organised a date for a telephone interview. Before the interview, the interviewer did not meet the interviewee. No other persons were present during the interviews. To begin the interview, an initial narrative question was asked: ‘Could you describe how you perceived the moment of receiving the diagnosis?’. After that, impulse giving guiding questions concerning quality of care were asked and if necessary, also deepening questions. See Additional File 1 for the overarching guiding questions (e.g. ‘To what extent has isPO met your individual needs?’) that were included in the interview guidelines to gain insight into patients’ care experiences. The interview guideline was developed by the external evaluation team. It was piloted with three cancer survivors from the project partner, House of the Cancer Patient Support Associations of Germany (HKSH-BV).

Data collection was conducted by the entire female evaluation team whose professional backgrounds included experiences and qualification from the areas of sociology, psychology, psychotherapy, nursing, public health, and health services research. Two team members hold a PHD degree, whereas the other two have a Master of Science degree and were in the process of obtaining their doctorate. Interviews were audio recorded and transcribed. Notes were taken during the interviews.

#### Qualitative analysis

Two members of the evaluation team were involved in data analysis using the software programme MAXQDA 2018 [50]. Qualitative content analysis was applied, which is a research question oriented and stepped systematic approach [51–53]. In the first interview wave, a coding system with core and sub-categories was developed deductively based on the interview lead questions. The two analysers then coded the transcripts independently using MAXQDA. In addition, inductive categories were derived from the material. The new categories were discussed to achieve a profound understanding of patients’ experiences, and the final category system was agreed upon. Then, new coding was carried out using the final category system. The content of the statements belonging to a category was condensed. For the second interview wave, the coding system of the first wave was used to analyse the material. In addition, inductive categories were derived. Again, categories were discussed, and a consensus was reached on a final category system, which was used for final coding. The same procedure was applied for the third interview wave. Data collection was conducted until rich data on patient’ experiences was obtained. As we focus on subjective meaning in data analysis, we refrained from quantifying the results [54]. However, we use phrasings like ‘some’, ‘all’, or ‘one patient’ to differentiate individual opinions and experiences from opinions of several or all interviewed patients.

## Results

### Quantitative results

In total, we contacted 1599 isPO patients from all four isPO care networks to participate in the first patient survey (T1), of which 62.2% (*n* = 994) completed and returned their questionnaire. All patients who finished their isPO care until the end of April 2021 and participated in the first survey (*n* = 867) were contacted for the second survey; 59.3% (*n* = 514) of patients participated in the second survey (T2).

Patients’ age ranged between 18 and 93 years (T1), with an average age of 56.88 years (T1). There were more female than male patients in the samples (T1: 64.7%, *n* = 637; T2: 67.8%, *n* = 345). Most patients were employed in the first survey sample (54.4%, *n* = 526), whereas slightly more patients were unemployed or retired in the second survey (50.8%, *n* = 248). Most patients were married or in a relationship (T1: 74.0%, *n* = 741; T2: 72.9%, *n* = 373). In the T1 sample, 58.05% of patients received care in Care Network 1 (*n* = 577), while patients from the other three care networks were represented with a rate of 10.16% (*n* = 100) up to 17.76% (*n* = 176). In the second survey (T2), 53.50% of patients (*n* = 275) received care in Care Network 1. Table 2 presents descriptive results on the predictors and outcome variables (please see Additional File 2 for frequencies of single items regarding satisfaction with care).

On average, patients rated their satisfaction with the different isPO service providers positively (Table 3). Satisfaction with care and orientation to needs was also rated positively, whereas rating of subjective effectiveness was slightly less positive (neutral to less satisfied). On average, frequency and duration of appointments were perceived as suitable.

**Table 3:** Descriptive analysis of variables

Variable	N	M	SD	Min	Max
Satisfaction with case management T1^a^ (health literacy-sensitive communication, HL-COM)	865	3.28	0.65	1.00	4.00
Satisfaction with the isPO onco-guide T1	692	3.39	0.60	1.00	4.00
Satisfaction with the psychosocial professional T2	252	3.16	0.46	1.00	4.00
Satisfaction with the psychotherapist T2 (therapeutic alliance, WAI)	200	3.88	0.89	1.00	5.00
Subjective effectiveness T2	409	2.90	0.74	1.00	4.00
Satisfaction and orientation to needs T2	415	3.27	0.68	1.00	4.00
Frequency of appointments T2	353	0.76	0.43	0	1.00
Duration of appointments T2	361	0.88	0.33	0	1.00
Global health status T1 (EORTC-QLQ-C30)	978	52.82	23.19	0	100.00
Global health status T2 (EORTC-QLQ-C30)	507	61.64	21.24	0	100.00
Work ability T1 (WAS)	956	3.69	3.05	0	10.00
Work ability T2 (WAS)	488	5.03	3.15	0	10.00
Anxiety and depression T0^b^ (HADS)	1752	16.31	8.70	0	42.00
Anxiety and depression T1 (HADS)	1316	14.18	8.25	0	41.00
Anxiety and depression T2 (HADS)	722	12.91	8.24	0	39.00

^a^patient surveys: T1 = 3 months into isPO care and T2 = at the end of isPO care (after 12 months)

^b^T0 = patient enrolment, T1 = 4 months into isPO care and T2 = at the end of isPO care (after 12 months)

For 423 isPO participants, data on global health status were available for both survey points. The mean value of the global health status is higher at T2 than at T1. Paired difference is significant according to the t-test for dependent samples: t(1422) = -7.353, *p* < 0.001, 95% CI [-9.36, -5.41]. Linear regression analyses show that, except for therapeutic alliance and items regarding temporal framework of care, all other predictors significantly and positively predict or associate with global health status (Table 4). Higher satisfaction with case management (regarding health literacy-sensitive communication), with isPO onco-guides and psychosocial professionals is associated with higher global health status at the end of care (T2). Furthermore, higher satisfaction with the scales 'subjective effectiveness' and 'satisfaction and orientation to needs' is associated with higher global health status (T2).

**Table 4:** Results of the regression analyses

Predictor	F (df regression, df residual)	Corr. R^2^	β	B	p	95% CI Min; Max
**Criterium variable: global health status (T2)**						
Satisfaction with case management T1 (HL-COM)	11.72 (1, 382)	.027	0.17	5.96	** < .001**	2.54; 9.39
Satisfaction with the isPO onco-guide T1	8.73 (1, 300)	.025	0.17	6.79	**.003**	2.27; 11.32
Satisfaction with the psychosocial professional T2	9.19 (1, 249)	.032	0.19	8.96	**.003**	3.14; 14.78
Satisfaction with the psychotherapist T2 (therapeutic alliance)	0.01 (1, 197)	-.005	-0.01	-0.19	.911	-3.59; 3.20
Subjective effectiveness T2	5.75 (1, 406)	.012	0.12	3.37	**.017**	0.61; 6.12
Satisfaction and orientation to needs T2	10.00 (1, 412)	.021	0.15	4.73	**.002**	1.79; 7.67
Frequency of appointments T2	2.94 (1; 350)	.005	0.09	4.51	.087	-0.66; 9.68
Duration of appointments T2	1.84 (1; 358)	.002	0.07	4.60	.176	-2.08; 11.28
**Criterium variable: work ability (T2)**						
Satisfaction with case management T1 (HL-COM)	8.39 (1, 371)	.019	0.15	0.76	**.004**	0.24; 1.28
Satisfaction with the isPO onco-guide T1	7.57 (1, 292)	.022	0.16	0.34	**.006**	0.27; 1.62
Satisfaction with the psychosocial professional T2	2.62 (1, 242)	.007	0.10	0.70	.107	-0.15; 1.55
Satisfaction with the psychotherapist T2 (therapeutic alliance)	3.59 (1, 192)	.013	0.14	0.49	.060	-0.02; 0.99
Subjective effectiveness T2	4.08 (1, 394)	.008	0.10	0.43	**.044**	0.01; 0.86
Satisfaction and orientation to needs T2	12.98 (1, 401)	.029	0.18	0.82	** < .001**	0.37; 1.27
Frequency of appointments T2	5.57 (1; 339)	.013	0.13	0.95	**.019**	0.16; 1.75
Duration of appointments T2	3.01 (1; 348)	.006	0.09	0.91	.084	-0.12; 1.93
**Criterium variable: anxiety and depression (T2)**						
Satisfaction with case management T1 (HL-COM)	17.06 (1, 469)	.033	-0.19	-2.32	** < .001**	-4.43; -1.22
Satisfaction with the isPO onco-guide T1	13.68 (1, 365)	.033	-0.19	-2.75	** < .001**	-4.22; -1.29
Satisfaction with the psychosocial professional T2	12.09 (1, 239)	.055	-0.24	-4.20	** < .001**	-6.33; -2.07
Satisfaction with the psychotherapist T2 (therapeutic alliance)	0.37 (1, 187)	-.003	0.04	0.39	.544	-0.87; 1.65
Subjective effectiveness T2	1.08 (1, 391)	.000	-0.05	-0.55	.300	-1.58; 0.49
Satisfaction and orientation to needs T2	4.87 (1, 396)	.010	-0.11	-1.23	**.028**	-2.33; -0.13
Frequency of appointments T2	12.21 (1; 335)	.032	-0.19	-3.42	** < .001**	-5.35; -1.50
Duration of appointments T2	3.56 (1; 344)	.007	-0.10	-2.43	.060	-4.97; 0.11

For 403 patients, data on work ability were available for both survey points. The mean value of work ability is higher at T2 than at T1. Paired differences is significant according to the t-test for dependent samples: t(402) = -8.11, *p* < 0.001, 95% CI [-1.48, -0.90]. Linear regression analyses show that, except for satisfaction with the psychosocial professionals, therapeutic alliance, and duration of appointments, all other predictors significantly and positively influence work ability (Table 4). Higher satisfaction with case management (regarding health literacy-sensitive communication) and with isPO onco-guides is associated with higher work ability at the end of care (T2). Furthermore, higher satisfaction with 'subjective effectiveness', 'satisfaction and orientation to needs', and frequency of appointments is associated with higher work ability (T2).

For 722 patients, data on anxiety and depression were available for all survey points. The mean value of HADS significantly decreased across all survey points over time (T0 to T1: t(681) = 6.96, *p* < 0.001; T1 to T2: t(681) = 2.83, *p* = 0.005; T0 to T2: t(721) = 8.37, *p* < 0.001). Linear regression analyses show that, except for satisfaction with therapeutic alliance, subjective effectiveness, and duration of appointments, all other predictors significantly and negatively influence anxiety and depression (Table 4). Higher satisfaction with case management (regarding health literacy-sensitive communication), with isPO onco-guides and psychosocial professionals is associated with lower anxiety and depression at the end of care (T2). Furthermore, higher satisfaction with 'satisfaction and orientation to needs' and frequency of appointments is associated with lower anxiety and depression (T2).

#### Qualitative results

**Fig. 3:**
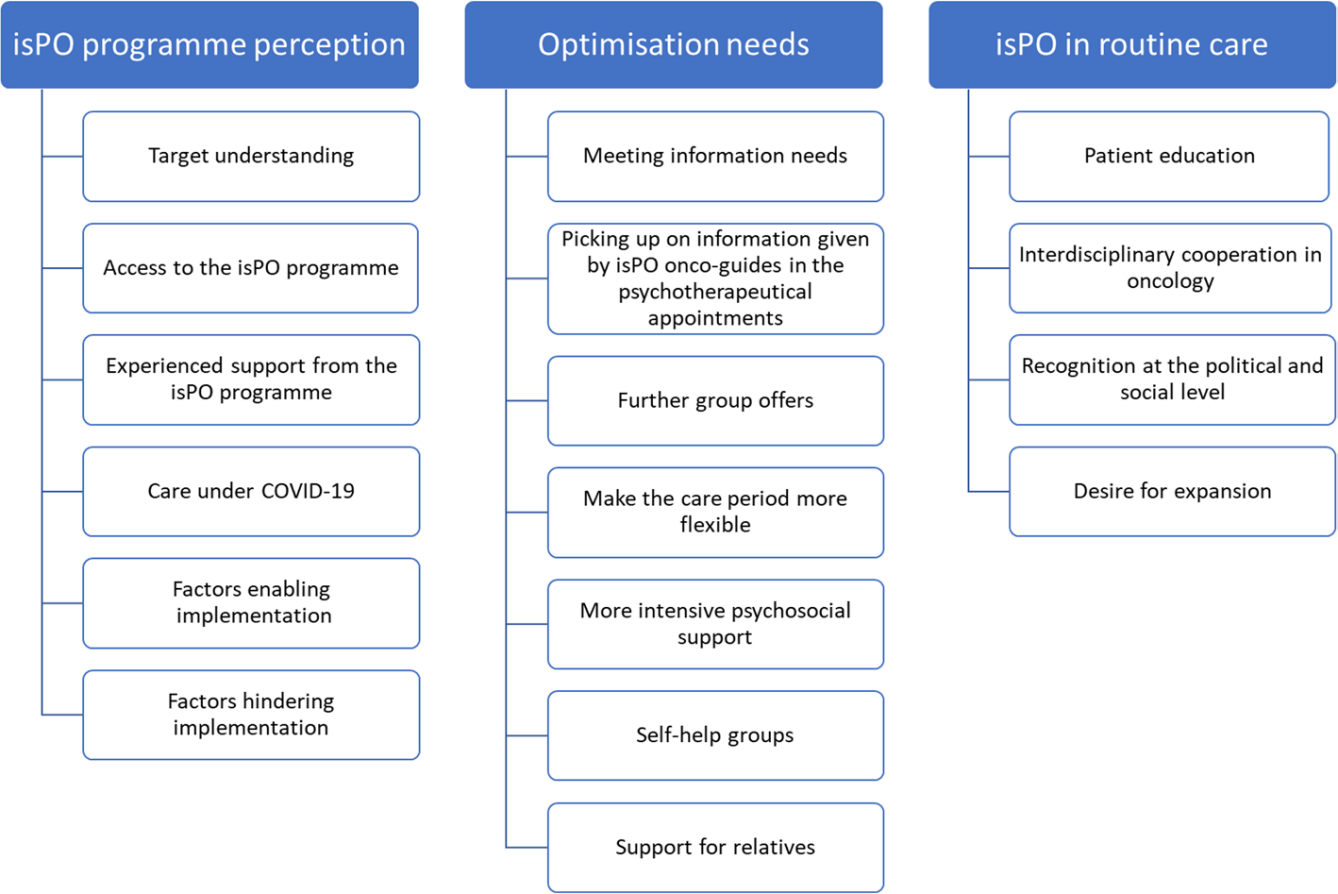
Head codings (first two levels) of the final coding system regarding quality of care in the isPO programme

Thirty-eight patients were approached for data collection, from which twenty-three agreed to participate. Reasons for not participating included feelings of emotional instability, ongoing cancer treatment or suffering from physical strains. Sixteen patients identified as female and seven male, and age ranged between 32 and 65 years. Seventeen patients were employed. The number and type of isPO care ranged between low-intensity to high-intensity care (see Additional File 3 for more details). All four isPO care networks and thirteen different cancer entities are represented in the data. The interviews took between 31 and 85 min. In all, the interview material comprises 21 h and 40 min. The final coding system consists of four levels. The head codings (first two levels) concerning quality of care are presented in Fig. 3.

Condensed results are first presented according to positive experiences and perceptions of the isPO programme, followed by negative perceptions and optimisation needs. Finally, patients’ attitudes towards isPO in routine care are described (see Additional File 4 for quotes).

#### Positive experiences and perceptions of the isPO programme

All interviewed patients perceived the isPO programme as useful for their individual recovery. Receiving PO care as a fixed care component parallel to medical oncological therapy appeared to be meaningful to them. The main goals of the isPO programme were identified by the patients as ‘*professional support’* and the patients being *‘closely supported’* which was considered important.

Patients’ access to the programme facilitated their programme acceptance. They found that ‘*being approached*’ was especially promising for enrolment. Most found it crucial that their treating physician (e.g. oncologist) approached and recommended programme enrolment and that isPO was offered in the same institution where the medical treatment took place (e.g. hospital). Timely access to care was considered necessary because it gave patients a feeling of security. Moreover, it had a supportive effect, as they had an obvious contact person for potential stressful experiences. However, it was argued that deciding to participate in the programme later should be ‘*handled flexibly*’.

Offering information and education on the programme at different levels (e.g. flyers, posters, in conversation with the case manager) with visual aids was considered motivating and necessary because ‘*a new programme like isPO is not self-explanatory*’ and PO is ‘*unknown to many patients and possibly has negative connotations*’.

Overall, patients felt that isPO supported them individually and that the content of the counselling, type of support, and the interprofessional work of the isPO service providers were valuable. The opportunity for outpatient care especially was perceived as crucial for securing their PO care. The timeframe of care (up to 12 months) and the flexible intensity of care, which is oriented towards the programme’s stepped care concept, was perceived as needs-oriented. It was perceived as valuable that sudden support needs could be flexibly addressed individually. Furthermore, some patients rated positively ‘*that there were hardly any waiting times*’ and that appointments were ‘*possible at short notice*’. They described the cross-sectoral continuity of care as a ‘*safety anchor*’. Fears often only arose after discharge from the hospital. Therefore, the continuity of care experienced in isPO from inpatient to outpatient setting and having a fixed contact person were considered helpful. This implied that patients knew that the isPO service providers were familiar with their individual case and that therefore, there was no information gap.

Within professional PO support, the formation of a good therapeutic alliance was essential, leading to a feeling of being ‘*understood, supported and cared for*’. The provision of information by the psycho-oncologists on further support options and the involvement of relatives was also positively received. Overall, patients described flexible handling of the care setting as helpful. Over the course of the project, the way in which the appointments were conducted became more flexible and adapted to the respective patient’s contextual circumstances. isPO was offered in different ways: (1) face-to-face in the rooms of the hospital itself, which was in line with the basic isPO idea, but also increasingly (2) by telephone, (3) online, or (4) via e-mail due to pandemic-related contact restrictions. However, it was emphasised that the first conversation should take place face-to-face, where possible.

Patients who utilised the low-threshold support offered by the isPO onco-guides, perceived it as ‘*very helpful*’ and complementary to their professional care in isPO (e.g. psychosocial or psychotherapeutical support). They appreciated that the programme enabled ‘*an encounter on a peer level*’ at a time when patients ‘*would most likely not have sought contact with self-help*’. Often, it was remarked that it ‘*felt good*’ to talk to a person who had ‘*gone through the same things*’. The interaction and communication on ‘*equal footing*’ felt ‘*liberating*’ and provided ‘*confidence and courage*’. Young patients especially found it helpful to be able to exchange experiences ‘*authentically*’.isPO service providers (e.g. isPO case management or psychologists) were perceived as very professional. Furthermore, the organisational professionalism (e.g. enrolment process with the health insurance companies, communication with the general practitioner at discharge) was highlighted. Here, isPO providers’ reachability was an important aspect, especially during the COVID-19 pandemic. The patients considered it reassuring that ‘*if there was a need to talk, you could always call, and someone would answer*’.

#### Negative perceptions of the isPO programme and optimisation needs

Patients articulated that the implementation of isPO was inhibited by the stigma attached to PO care in Germany. In their opinion, there has not yet been a sufficient cultural change in society, and this is especially true for the older population. Directly related to this is the obstacle of explaining the programme coherently. As the isPO programme is complex, most patients find it difficult to understand it in detail, which may lead to programme rejection. This was especially noticeable in the interview data of the first two interview waves (early implementation phase). It improved with the programme’s progress, presumably when the care networks began to use optimised patient information materials (PIMs). In the third wave of interviews, after the optimised PIMs were utilised in all networks, patients no longer described any obstacles in this regard.

Similarly challenging was the comprehensibility of the isPO onco-guide concept. Especially at the beginning of the implementation (first wave data), it was noticeable that patients expressed little need to make use of the isPO onco-guides. It became clear that this was mostly because the isPO service providers did not provide accurate information about the onco-guides' duty and role, which in turn led to misunderstanding. Furthermore, some patients were reluctant to meet with a onco-guide out of ‘*fear of being overloaded with other bad stories*’ or a ‘*desire for peace*’. However, some patients developed an openness to meet with an isPO onco-guide later in the course of their trajectory. During the pandemic, some patients refused the offer due to fears of infection with COVID-19 through face-to-face contact. Furthermore, the resource structure of the isPO onco-guides was perceived as partially hindering. In some settings, rooms for a *‘sensitive conversation’* were not available and they unfortunately ‘*had to move to the cafeteria*’. At the time of the COVID-19 pandemic, there were also ‘*bottlenecks in terms of staff*’, as isPO onco-guides also feared infection.

Patients experienced that staff (e.g. physicians, nurses) in the oncological wards only knew little about the isPO care programme. Furthermore, the external marketing for the isPO programme was described by some patients as ‘*insufficient*’ and ‘*hardly available*’.

Overall, many of the interviewed patients perceived the care period as not flexible enough. The abrupt end after 12 months was seen by some patients as ‘*questionable*’ because many patients were still undergoing medical therapy at this time; therefore, many might still have needed the support. They desired structured PO aftercare.

Some patients had difficulty differentiating between the care options provided by isPO and other outpatient psychotherapeutic treatment services. This may have led to different expectations of patients and service providers in relation to the content of care. In addition, some patients confused the programme and study contents, which is probably due to the interconnection of programme and study during the project period. Some described this as *‘frustration*’ because the meaning of the different questionnaires was not clear to them. With the new PIMs (e.g., timeline), this perception was minimised and was no longer described in the final interviews.

Further programme optimisation included the desire for the expansion of psychosocial and family support because ‘*cancer is a “we” disease*’.

#### isPO in routine care

All interviewed patients expressed a desire for the ‘*availability of isPO after the end’* of the project phase and the expansion of the programme ‘*for all cancer patients*’. They emphasised that this would require a cultural change in the perception of psycho-oncology in Germany. Lack of knowledge and stigma might lead to aversion towards PO care. One patient described it as a ‘*rethinking in society*’ that needs to take place. For this aim, ‘*comprehensive and continuous education*’ and marketing (e.g. through display walls, posters, radio, or advertisements) could be used.

From the patients' point of view, local and nationwide implementation of PO care is only possible with a constant expansion of personnel resources, sufficient qualification opportunities for staff, and balanced financial resources. It is therefore important that sufficiently qualified psycho-oncologists are available for needs-based PO care (i.e. that staff positions are created). One patient understood the problem like this: ‘*It's no use, if I offer this and don't have the staff to take care of it in peace. […] and you only have one contact person. So he is hopelessly overworked and that doesn't help either.*’

Patients emphasised that long-term and continuous PO support provided stability and was therefore highly relevant. An ‘*abrupt end’* to PO counselling after inpatient medical treatment should be avoided, and continuation of needs-oriented care, such as in isPO for 12 months, is desirable. Several of the interviewed patients identified the need for sustainable structures to achieve this purpose. They expressed the need for ‘*every hospital that treats oncological patients’* to offer PO services in a way that is financed and can therefore be expanded and/or maintained. Furthermore, some patients called for a good interdisciplinary cooperation between oncology and psycho-oncology to enable comprehensive cancer treatment.

A few patients expressed the need for better visibility and knowledge about PO care within the health system, for example, among general practitioners. In their opinion, communication about available support programmes should be enhanced through professional articles in journals and through presentations of the study results of programmes like isPO.

The interviewed patients showed awareness that the described aspects are a societal and cultural task that can only be implemented through recognition at the political and societal levels and through measures (implementation of guidelines) and the commitment of all those involved. From the patients’ point of view, different but focused actions (e.g. information week for psycho-oncology, advertisements) might be necessary.

## Discussion

### Quality of care in isPO

Within both methodological approaches (quantitative and qualitative), patients reported medium-to-high satisfaction with the care they received in isPO. Furthermore, we found significant improvements in patient-related outcomes (health status, work ability, and anxiety and depression) over time, and regression analyses indicate that satisfaction with quality of care influenced those outcomes. Already, low-threshold care provision (e.g. via isPO case management, isPO onco-guides, and/or psychosocial care) significantly and positively impacts these patient outcomes, which is in line with guidelines that recommend stepped and needs-oriented PO care [55]. However, in our analyses, it is noticeable that therapeutic alliance (regarding psychotherapeutic care) does not show significant association with the outcome variables. We presume an indirect effect, with therapeutic alliance significantly affecting general care satisfaction (subjective effectiveness and needs orientation) [56], which in turn affects important patient related outcomes. Furthermore, satisfaction with the frequency of appointments positively affected patients’ work ability, anxiety, and depression. This underscores the importance of care continuity and outpatient care and might be of interest for health insurance companies for economic reasons. Patients who return to work earlier might financially relieve health insurance companies in the long term. Research based on German health insurance claims data revealed that psychotherapy significantly reduced care costs and days of incapacity to work (i.e. sick days) [57–59]. Wittmann et al. [60] even suggested that ‘every euro invested in outpatient psychotherapy’ pays off threefold for society under the premise that the therapy effect lasts for 1 year after the end of treatment. In addition, patients have reported that returning to work is an important aspect for a full recovery [61, 62]; it positively affects quality of life [62, 63] and provides financial security [62, 64] and a sense of control [65, 66]. Therefore, needs-oriented, continuous outpatient PO care may help patients reach a level of functioning that enables them to return to work and improve their quality of life.

The qualitative results allowed us to gain deep insights into patient experiences with isPO. Moreover, they represent the specific enablers and barriers to programme implementation and therefore also quality of care. Patients found outpatient care with a fixed contact person crucial for care continuity; this was also promoted by Fann et al. [67]. The patients’ experiences highlight that needs-oriented care was not just achieved by allocating patients to the ‘right’ care level based on sole occurrence of symptoms; it was also connected to increased flexibility in care that took patients’ individual needs into consideration [55, 68, 69]. For example, this was achieved by flexibly choosing the care delivery mode (e.g. phone, face-to-face, or virtual) and the appointment frequency. Our findings on satisfaction with appointment frequency aligns with further external isPO evaluation results, indicating that the number of appointments with psychotherapists (utilised by patients with higher care needs) significantly influence changes in anxiety and depression over time [70]. Therefore, needs-orientation seems to be a key component in PO care.

Patients identified other aspects that could be handled more flexibly in the care programme: time of programme enrolment, period of care, and extension of care to relatives. The patients’ relatives’ need for care is often neglected [71], even though they may also suffer from emotional and social impairments [72, 73]. Therefore, from a patient’s perspective, it may be recommendable to augment isPO with a component that aims to flexibly address their relatives’ support needs.

### Implications for quality of care in psycho-oncology

Measuring patient-reported outcomes may enhance patient-centred care and be beneficial for clinical outcomes [74–76]. The isPO programme endeavoured not only to close the healthcare gap of needs-oriented cross-sectoral PO care, but also to establish a structured, sustainable healthcare programme that includes an adequate quality management. Through external evaluation of the isPO programme, it became evident that it is crucial to include patients’ perspectives on quality of care. Investing in gathering patients’ perspectives offered the opportunity to gain specific and practice-relevant feedback on important optimisation needs and implementation enablers and barriers that were also experienced by other researchers [77]. Patients provided feedback specific to the implementation site (e.g. medical personnel not knowing about the isPO intervention programme) in addition to general feedback (e.g. that the care period needs to be handled flexibly according to the patients’ needs). Therefore, structured quality management at each care site and site-overarching (e.g. benchmarking or quality workshops) should be implemented and maintained to facilitate patient engagement in their care reality.

Perceiving and considering patients’ perspectives should be acknowledged as an important quality indicator for needs-oriented interventions [78, 79]. For the daily routine of quality management, specific PO quality indicators are required to sufficiently assess, monitor, and improve quality of care [55, 80]. Breidenbach et al. [81] came to a similar conclusion; they exploratorily analysed audit data of cancer centres regarding the challenges of providing PO care and found diverse care barriers on both the patient and organisational level. They called for the identification and integration of processual measures that especially promote integrated PO care in routine oncological care [81]. This aligns with Rubin et al.’s [79] reported advantages of integrating process measures of quality. They highlighted that implementation of such quality indicators may empower service providers and clinicians to proactively influence patient-reported outcomes.

Based on patients’ experiences in isPO, we formulated recommendations for the programme, especially regarding an adaptability to routine care (Table 5). Furthermore, patients expressed diverse attitudes and recommendations that apply to PO care in general.

**Table 5:** Recommendations for the isPO programme and psycho-oncological care in Germany based on cancer patients’ care experiences

**Recommendations regarding the isPO programme**	
Needs-oriented patient support	Allow flexibility regarding the timing (start and end of care) → *Patients have different needs, emotional coping mechanisms, and illness severity.*
	Maintain flexibility in the delivery of care (mode and frequency of care delivery) * → patients have different preferences and access to care. Hence, care delivery that allows appointments to be conducted flexibly face-to-face, via telephonic or videochat, may facilitate needs-oriented care. The same applies for the frequency of appointments, which can vary between patients due to different care needs and cancer treatment* → *It promotes needs-oriented care and makes care structures more adaptable to disruptions in the healthcare system (e.g. pandemic).*
Staff education and information flow	Provide adequate training (initial and ongoing) of isPO service providers and medical personnel towards the new programme and promote cooperation between oncological and psycho-oncological services → *Lack of knowledge (e.g. oncologists not knowing about the programme) or inadequate information (e.g. case managers knowledge regarding the role of isPO onco-guides) may impede high-quality programme delivery and accessibility for patients. Good cooperation and medical staff’s acceptance of psycho-oncological care may reduce barriers in patients’ access to care.*
	Address information loss due to staff rotation within the oncological departments → *Regular, periodic information sessions would be helpful to maintain the information flow (e.g. newsletters or information sessions).*
New programme elements	Reflect on the integration of further psycho-oncological care services → *Severe illnesses may affect not just the patient, but also their family and social environment. Expanding care to relatives may be helpful. Some patients require specific support, such as art therapy. Providing suitable offers might elementarily support these patients.*
**Recommendations regarding routine psycho-oncological care**	
Cultural change	Invest in reducing stigma surrounding utilisation of psycho-oncological care → *Using a multilevel approach (e.g. *via* media, peer groups, and good practice examples) is considered helpful.*
Patient information and education	Use end user-friendly patient information material → *It may give patients orientation, e.g. on psycho-oncological care processes or important contact persons to access care services, and satisfy information needs, e.g. in regard to what psycho-oncology is, comprehensibly. Further, user-friendly material may facilitate informed decision-making.* → *If new materials need to be designed, consider the inclusion of patients’ experiences and perspectives (empowerment) *[82].
Sustainability and multidisciplinary	Implement structured financing for cross-sectoral psycho-oncological care → *Patients emphasised the need to ensure needs-oriented and structured care models, such as isPO, by assuring that they receive sufficient financing. The German healthcare system omits financing of psycho-oncological care that is structured and anchored in law. Investing in psycho-oncological care may be beneficial for insurance companies in the long term (e.g. in terms of increasing work ability)*
	Implement interlocking, multidisciplinary structured needs-oriented care (e.g. isPO) → *Patients expressed that multidisciplinary teams facilitated needs-oriented care; interlocking them simplified access to respective care services*
	Avoid sectoral separation of care as patients desire care continuity with fixed contact persons (in- and outpatient)

### Methodological strengths and limitations

Applying a mixed methods design is characterised as key to contextualising patient experiences in health care [83]. It allowed us to use the strengths of both, quantitative and qualitative methodologies and offset their respective weaknesses [84, 85] Further, it provided a deep understanding of patients’ perspective on the complex isPO intervention programme and helped to include their perspectives early and continuously in the programme’s optimisation loops [82, 86]. Participative elements in a programme’s development have been considered helpful by other researcher also [32, 87–89]. Programme designers in isPO predominantly used a top-down approach during the development phase [31]. However, by feedbacking our results (‘acute’ results were immediately articulated) at least once a year to the programme designers, programme optimisations could be initiated by the designers according to the end-user’s needs [35, 37]. This may make the programme more adaptable and tailored for routine care [89]. Therefore, formative evaluation data on quality of care may function as an indicator for the maturity level of a complex intervention and may aid in early identification of strengths and weaknesses that are specific to the implementation setting [90]. In addition, it is important to consider though that a comprehensive mixed methods design needs sufficient resources and adequate funding.

Even though an exploratory sequential design might have been helpful for a broader generalisation of the results [91], the explanatory design allowed us to explain the quantitative results and gather in-depth information on quality of care. Moreover, the qualitative approach may address some of the limitations of patient-reported experiences, such as confounding by health outcomes or measuring expectations rather than actual experiences [92].

Our quantitative data suggest that processual quality of care measurements affect patient-reported outcomes, also the primary outcome of isPO (anxiety and depression). Therefore, we promote that, during evaluation of a new complex interventions, quality of care should be considered an important study outcome, which was also pronounced by others [93, 94]. Furthermore, when assessing patient-reported outcomes, there is a risk for biases, such as social desirability, common method, or recall. However, at the same time, patient-reported outcomes are valuable indicators of quality of care. Given the absence of a control group, conclusions regarding causality may not be drawn. The synthesis of results on quality of care together with the results on a programmes’ effectiveness is therefore important when considering the complexity of a new form of care, like isPO (effectiveness results will be published elsewhere).

Interviewing patients after they finished 1 year of care in isPO was additionally helpful as they were able to reflect on the entire isPO care trajectory. Considering different moments (e.g. early implementation) within the implementation process allowed us to observe the programme normalisation process in the different care networks, which was also promoted by May and colleagues [95]. However, as the patient recruitment process for the interviews was initiated by service providers, this should be considered when interpreting the results. We might have an underrepresentation of patients who were (1) not satisfied with isPO, (2) timid, or (3) critically affected by oncological treatment. However, most patients we interviewed did not refrain from giving feedback on possibilities for optimisation. Finally, only patients who were mostly fluent in German were recruited for the interviews. Further studies exploring the needs of patients with limited language proficiency are indicated.

## Conclusions

Patients assess the isPO programme’s quality of care positively. Likewise, patients’ perspectives were crucial for identifying implementation enablers and barriers of this new form of PO care, reflecting the programme’s feasibility and possible fit for routine care. Our results suggest a positive relationship between patients’ satisfaction with quality of care and important patient-related outcomes (health status, work ability, anxiety, and depression). Therefore, investing in gathering data on patients’ perspectives while using a mixed methods design can be helpful for conducting comprehensive evaluation of complex interventions to assess their quality of care and thereby maturity. For designers, this data may support necessary programme optimisations, especially if participative elements were not considered in the project’s design phase [96]. Even though the isPO programme is highly complex, with its various interacting programme components [22], patients’ experiences with the stepped and needs-centred care approach indicate that it is recommendable for routine care. However, the persistent programme optimisation should be conducted and integrated within structured quality management.

## Supplementary Information

**Additional file 1: Table A.** Impulse giving guiding questions of the interview guidelines concerning quality of care.

**Additional file 2: Table B.** Frequencies of variables assessing patients’ satisfaction with their respective service providers and the isPO care in general

**Additional file 3: Table C.** Characteristics of interviewed patients (table content first published in Krieger et al. 2022).

**Additional file 4: Table D.** Exemplary quotations for the coding system.

## Data Availability

The datasets generated and analysed during the current study are not publicly available due to participants’ consent restricting data use to the research team, but are available from the authors (corresponding author and/ or holger.pfaff@uk-koeln.de) on reasonable request. Due to German data protection law, only de-identified data may be shared.
