# Supplementary material for: Patients’ perspectives on the quality of care of a new complex psycho-oncological care programme in Germany – external mixed methods evaluation results
Source: BMC Health Serv Res. 2023 Jul 15;23:759. doi: 10.1186/s12913-023-09714-y (PMC10349427; doi:10.1186/s12913-023-09714-y)
Supplement: Supplementary file 1 — Additional file 1: Table A. Impulse giving guiding questions of the interview guidelines concerning quality of care. [file 12913_2023_9714_MOESM1_ESM.docx]

**SUPPLEMENTARY MATERIAL**

**Additional file 1**

**Table A**. Impulse giving guiding questions of the interview guidelines concerning quality of care

| **First interview wave** | **Second interview wave*** | **Third interview wave** |
| --- | --- | --- |
| How would you describe isPO to a friend? | What experiences have you had in your isPO care? | How would you describe isPO to a friend? |
| The isPO programme aims to offer support to patients depending on their individual needs. For this, the patient can theoretically come into contact with several people.  What was it like for you? In what way have you received support within the isPO programme? | What experiences have you had with the treating isPO team? | The isPO programme aims to offer support to patients depending on their individual needs. For this, the patient can theoretically come into contact with several people.  What was it like for you? In what way have you received support within the isPO programme? |
| The isPO programme was newly introduced at the XX hospital, which means that the hospital had to introduce new structures and procedures.  Did you ever feel that these changes showed in contact with you? | Looking back on your care in isPO, would you recommend it to other cancer patient? | To what extent did organisational challenges arise during your care process? |
| How professional did you perceive the different isPO service providers? | How have you experienced isPO care since the Corona pandemic, since March 2020? | Looking back on the 12 months of support in the isPO programme during the pandemic. To what extent did isPO meet your individual support needs? |
| To what extent has isPO met your individual needs? | What did you particularly like about the isPO programme? | Looking back, what has been your personal value from participating in the isPO programme? |
| What did you particularly like about the isPO programme? | In isPO, you were able to receive support for 12 months after diagnosis.  How did you feel about the 12-month period? | In isPO, you were able to receive support for 12 months after diagnosis.  How did you feel about the 12-month period? |
| In isPO, you were able to receive support for 12 months after diagnosis.  How did you feel about the 12-month period? | What did you find unusual?  What did you find unpleasant or in need of improvement? | What did you find unusual?  What did you find unpleasant or in need of improvement? |
| What did you find unusual?  What did you find unpleasant or in need of improvement? | isPO is a novel programme that has only been offered and tested at 4 hospitals.  What would you wish isPO for the future?  (added subquestion: What would be necessary to offer isPO nationwide?) | isPO is a novel programme that has only been offered and tested at 4 hospitals.  What would you wish isPO for the future?  (subquestion: What would be necessary to offer isPO nationwide?) |
| Looking back, would you say that participating in the isPO programme was worthwhile for you personally? |  |  |
| isPO is a novel programme that has only been offered and tested at 4 hospitals.  What would you wish isPO for the future? |  |  |
| *the guideline of the second interview wave was adapted to the extent that a project cooperation partner could use the data for the programme’s internal evaluation process. By this, it was avoided to overload patients and care networks with interview requests. | | |
