# Supplementary material for: Patients’ perspectives on the quality of care of a new complex psycho-oncological care programme in Germany – external mixed methods evaluation results
Source: BMC Health Serv Res. 2023 Jul 15;23:759. doi: 10.1186/s12913-023-09714-y (PMC10349427; doi:10.1186/s12913-023-09714-y)
Supplement: Supplementary file 2 — Additional file 2: Table B. Frequencies of variables assessing patients’ satisfaction with their respective service providers and the isPO care in general [file 12913_2023_9714_MOESM2_ESM.docx]

**Additional file 2**

**Table B**. Frequencies of variables assessing patients’ satisfaction with their respective service providers and the isPO care in general

| **Items** | | | **N** | | I don’t agree at all  **% (N)** | | I rather don’t agree  **% (N)** | | I rather agree  **% (N)** | | | I totally agree  **% (N)** | |
| --- | --- | --- | --- | --- | --- | --- | --- | --- | --- | --- | --- | --- | --- |
| **isPO-case management (T1)** | | | | | | | | | | | | | |
| I was taught that it was important for me to understand the information about my condition and treatment. | | | 913 | | 3.3 (30) | | 9.2 (91) | | 38.0 (347) | | | **48.7** (445) | |
| I was asked whether I understood information or documents. | | | 915 | | 3.5 (32) | | 10.5 (96) | | 31.1 (285) | | | **54.9** (502) | |
| Verbal information about my illness or treatment was also given to me in writing. | | | 898 | | 7.2 (65) | | 17.8 (160) | | 31.2 (280) | | | **43.8** (393) | |
| Technical terms and abbreviations were explained to me during the discussions. | | | 904 | | 7.4 (67) | | 19.8 (179) | | **40.9** (370) | | | 31.9 (288) | |
| In the conversations, people spoke clearly and slowly enough. | | | 909 | | 1.5 (14) | | 6.4 (58) | | 35.1 (319) | | | **57.0** (518) | |
| I was encouraged to ask questions when I didn't understand something. | | | 908 | | 2.9 (26) | | 8.5 (77) | | 28.0 (254) | | | **60.7** (551) | |
| Written information was additionally explained to me verbally. | | | 901 | | 6.4 (58) | | 18.0 (162) | | 37.0 (333) | | | **38.6** (348) | |
| With consent forms, emphasis was placed on making sure that I really understood everything. | | | 909 | | 3.6 (33) | | 9.5 (86) | | 37.0 (336) | | | **49.9** (454) | |
| **isPO onco-guide (T1)** | | | | | | | | | | | | | |
| The information I received from the onco-guide was helpful for me. | | | 717 | | 2.5 (18) | | 9.2 (66) | | 41.4 (297) | | | **46.9** (336) | |
| The onco-guide behaved in a way that I could feel comfortable. | | | 712 | | 2.7 (19) | | 3.9 (28) | | 26.1 (186) | | | **67.3** (479) | |
| The onco-guide was able to answer my questions satisfactorily. | | | 706 | | 2.3 (16) | | 7.6 (54) | | 36.5 (258) | | | **53.5** (378) | |
| The onco-guide had too little time for me. | | | 714 | | **59.9** (428) | | 26.5 (189) | | 8.1 (58) | | | 5.5 (39) | |
| I felt connected to the onco-guide. | | | 708 | | 4.4 (31) | | 15.7 (111) | | **49.6** (351) | | | 30.4 (215) | |
| Overall, I was satisfied with the onco-guide talk. | | | 715 | | 2.7 (19) | | 7.6 (54) | | 35.1 (251) | | | **54.7** (391) | |
| **Psychosocial professional (T2)** | | | | | | | | | | | | | |
| I received useful information during counselling. | | | 271 | | 1.2 (4) | | 5.9 (16) | | 43.9 (119) | | | **48.7** (132) | |
| The social worker [psychosocial professional] answered all my questions. | | | 264 | | 3.0 (8) | | 5.3 (14) | | 34.5 (91) | | | **57.2** (151) | |
| The counselling sessions were too short. | | | 268 | | **40.7** (109) | | 35.4 (95) | | 18.7 (50) | | | 5.2 (14) | |
| The social worker [psychosocial professional] took my personal circumstances and environment into account. | | | 249 | | 3.0 (8) | | 7.2 (19) | | 42.2 (111) | | | **47.5** (125) | |
| The social worker [psychosocial professional] advised me in such a way that I was able to implement advice and assistance in a concrete way. | | | 248 | | 4.2 (11) | | 7.2 (19) | | 42.8 (113) | | | **45.8** (121) | |
| **Psychotherapist (T2)** | | | | | | | | | | | | | |
| **Items** | **N** | | Seldom  **% (N)** | | | Sometimes  **% (N)** | | Often  **% (N)** | | Very often  **% (N)** | | | always  **% (N)** |
| My therapist and I respect each other. | 277 | | 4.7 (11) | | | 2.1 (5) | | 6.4 (15) | | 16.6 (39) | | | **70.2** (165) |
| I feel that my therapist appreciates me. | 232 | | 4.7 (11) | | | 3.4 (8) | | 8.6 (20) | | 17.7 (41) | | | **65.5** (152) |
| I fell my therapist care about me even when I do things that he/she does not approve of. | 212 | | 7.5 (16) | | | 6.6 (14) | | 17.5 (37) | | 30.2 (64) | | | **38.2** (81) |
| I feel that the things I do in therapy will help me to accomplish the changes that I want. | 221 | | 5.4 (12) | | | 10.4 (23) | | 21.3 (47) | | **45.2** (100) | | | 17.6 (39) |
| What I am doing in therapy gives me new ways of looking at how I am dealing with my disease. | 230 | | 8.7 (20) | | | 20.0 (46) | | 29.1 (67) | | **35.7** (82) | | | 6.5 (15) |
| My therapist and I collaborate on setting goals for my therapy. | 229 | | 13.1 (5.9) | | | 10.0 (25) | | 21.0 (48) | | **32.3** (74) | | | 22.7 (10.2) |
| My therapist and I have established a good understanding of the kind of changes that would be good for me. | 221 | | 5.4 (12) | | | 4.1 (9) | | 20.8 (46) | | **43.9** (97) | | | 25.8 (57) |
| I believe my therapist likes me. | 218 | | 5.0 (11) | | | 6.0 (13) | | 12.8 (28) | | 26.6 (58) | | | **49.5** (108) |
| My therapist and I are working towards mutually agreed upon goals. | 225 | | 8.4 (19) | | | 5.8 (13) | | 14.7 (33) | | 31.1 (70) | | | **40.0** (90) |
| My therapist and I agree on what is important for me to work on. | 228 | | 4.8 (11) | | | 4.4 (10) | | 10.5 (24) | | 38.6 (88) | | | **41.7** (95) |
| As a result of these sessions I am clearer as to how I might be able to change. | 285 | | 9.3 (21) | | | 8.4 (19) | | 25.6 (58) | | **35.2** (80) | | | 21.6 (49) |
| I believe the way we are working on how I can deal with my cancer disease is correct. | 221 | | 4.1 (9) | | | 5.9 (13) | | 14.9 (33) | | 36.7 (81) | | | **38.5** (85) |
| **Temporal framework conditions (T2)** | | | | | | | | | | | | | |
| Frequency of appointments was… | 353 | | Too rare:  %=23.8 N=84 | | | | Exactly right:  **%=76.2** N=269 | | | | Too often:  N=0 | | |
| Duration of appointments was… | 361 | | Too short:  %=11.9 N=43 | | | | Exactly right:  **%=87.3** N=317 | | | | Too long:  %=.03 N=1 | | |
| **Subjective effectiveness (T2)** | | | | | | | | | | | | | |
| **Items** | | **N** | | I don’t agree at all  **% (N)** | | | I rather don’t agree  **% (N)** | | | I rather agree  **% (N)** | | I totally agree  **% (N)** | |
| I feel better because of the care in isPO. | | 426 | | 5.2 (22) | | | 19.5 (83) | | | **45.8** (195) | | 29.6 (126) | |
| Care in isPO helped me deal with my illness. | | 426 | | 4.5 (19) | | | 15.5 (66) | | | **50.7** (216) | | 29.3 (125) | |
| Care in isPO increased my quality of life. | | 419 | | 6.3 (26) | | | 31.0 (130) | | | **44.4** (186) | | 18.4 (77) | |
| Care in isPO helped me change my attitude towards the disease. | | 422 | | 6.2 (26) | | | 24.4 (103) | | | **48.3** (204) | | 21.1 (89) | |
| **Satisfaction and Needs-orientation (T2)** | | | | | | | | | | | | | |
| I was satisfied with the care in isPO. | | 440 | | 3.0 (13) | | | 8.2 (36) | | | **45.0** (198) | | 43.9 (193) | |
| Overall, I found isPO helpful. | | 437 | | 3.0 (13) | | | 8.2 (36) | | | 42.3 (185) | | **46.5** (203) | |
| The care in isPO supported me exactly as I needed it according to my needs. | | 423 | | 5.4 (23) | | | 13.0 (55) | | | **52.2** (221) | | 29.3 (124) | |
| I would choose isPO again. | | 433 | | 5.1 (22) | | | 7.4 (32) | | | 32.6 (141) | | **55.0** (238) | |
