# Supplementary material for: Patients’ perspectives on the quality of care of a new complex psycho-oncological care programme in Germany – external mixed methods evaluation results
Source: BMC Health Serv Res. 2023 Jul 15;23:759. doi: 10.1186/s12913-023-09714-y (PMC10349427; doi:10.1186/s12913-023-09714-y)
Supplement: Supplementary file 3 — Additional file 3: Table C. Characteristics of interviewed patients (table content first published in Krieger et al. 2022). [file 12913_2023_9714_MOESM3_ESM.docx]

**Additional file 3**

**Table C**. Characteristics of interviewed patients (table content first published in Krieger et al. 2022)

| **Nr** | **isPO Care network*** | **Gender** | **age** | **Employment status** | **Cancer Entity** | **care stage**** | **Number and type of care** |
| --- | --- | --- | --- | --- | --- | --- | --- |
| **First interview wave (April-July 2020)** | | | | | | | |
| 1 | 1 | male (m) | 64 | retired | Rectal | 2 | 4 appointments with a psychoscoial professional (PP),  1 appointment with an isPO-onco guide (isPO-OG) |
| 2 |  | m | 39 | employed | Melanoma | 2 | 10 PP,  1 isPO-OG |
| 3 |  | female (f) | 60 | unemployed | Breast | 3a | 10 psychotherapeutical appointments (PT)  1 isPO-OG |
| 4 |  | f | 53 | employed | Small intestine | 3b | 6 PT  1 isPO-OG |
| 5 | 2 | f | 47 | employed | Bronchia | 3b | 5 PT |
| 6 | 3 | f | 52 | employed | Rectal | 3b | 25 PT |
| 7 |  | f | 50 | employed | Breast | 3b | 27 PT 3 PP |
| 8 | 4 | f | 52 | employed | Breast | 3b | 8 PT |
| 9 |  | f | 56 | employed | Breast | 3a | 8 PT |
| **Second interview wave (November 2020-March 2021)** | | | | | | | |
| 10 | 1 | m | 57 | Early retirement | Prostate | 2 | 4 PP |
| 11 |  | f | 32 | student | Thyroid | 3a | 7 PT |
| 12 |  | m | 54 | employed | Parotid | 3a | 10 PT |
| 13 |  | f | 55 | employed | Non-Hodgkin- Lymphoma | 3b | 6 PT |
| 14 | 2 | m | 65 | retired | Squamous epithelium | 3b | 5 PT |
| 15 |  | f | 51 | employed | Breast | 3b | 3 PT |
| 16 | 3 | f | 39 | Minor employment | Cervix | 3b | 19 PT |
| 17 |  | m | 63 | Early retirement | Appendix | 3b | 32 PT  2 PP |
| 18 | 4 | m | 61 | employed | Prostate | 2 | 4 PP |
| 19 |  | f | 41 | employed | Thyorid | 3a | 14 PT |
| **Third interview wave (April-June 2021)** | | | | | | | |
| 20 | 1 | f | 33 | employed | Breast | 3b | 12 PT |
| 21 | 2 | f | 58 | employed | Malignant neoplasm without indication of localisation | 3b | 14 PT |
| 22 | 3 | f | 36 | employed | Breast | 3b | 7 PP |
| 23 | 4 | f | 63 | employed | Kidney | 3b | 11 PT |
| *isPO care networks are pseudonymised from 1 to 4 to maintain data protection for patients and care networks. **care stages mean isPO care stages which range from 1 to 3b. They are oriented on patients’ individual needs (anxiety, depression, psychosocial problems and information needs) | | | | | | | |
