# Supplementary material for: Patients’ perspectives on the quality of care of a new complex psycho-oncological care programme in Germany – external mixed methods evaluation results
Source: BMC Health Serv Res. 2023 Jul 15;23:759. doi: 10.1186/s12913-023-09714-y (PMC10349427; doi:10.1186/s12913-023-09714-y)
Supplement: Supplementary file 4 — Additional file 4: Table D. Exemplary quotations for the coding system. [file 12913_2023_9714_MOESM4_ESM.docx]

**Additional file 4**

**Table D**. Exemplary quotations for the coding system.

| **Head code** | **Sub code** | **Exemplary quotations** |
| --- | --- | --- |
| isPO programme perception | Target understanding | *It's a programme which has enabled me to have psycho-oncological support during my illness and thereby also to have the opportunity to be closely supported.* |
|  | Access to the isPO programme | *I was very grateful that he [treating doctor] approached me so proactively, because I probably wouldn't have asked that question on my own at that point.*  *The case manager came to my room at some point, introduced herself [...] and we started talking. I thought it was good and wanted to join in.*  *It was a good thing that I had them the day after I was diagnosed. [...] So it gave me a very good feeling of security* |
|  | Experienced support from the isPO programme | *The opportunity to get care here, not so far from where I live, I thought that was great too.*  *I can imagine that maybe other relatively young cancer patients feel the same way. It is difficult when you are in your early 30s and look healthy. It is important to somehow give this disease space. [...] I think the principle of the onco-guide worked well for me, because I really didn't get any clever advice, which I would have found unpleasant. There was also nothing judgmental about it, but simply a lot of concentration, openness and presence. At that moment it was just right.*  *I never had the feeling that it was time for stress here because the next person was already coming in, but I always had the feeling: now you are the one who is being helped here and there is 100 percent time for me here.*  *The psycho-oncologist actually played my cards very well and helped me. She also looked after my relatives intensively and actually helped me as a result.* |
|  | Care under Corona | *Well, because of Corona we just agreed to do it on the phone and that worked out very well, didn't it? Sometimes I wasn't feeling so well after chemo either. But I always had the possibility to call if I needed help.*  *So, when you see eye to eye, of course it's different than on the phone. That's for sure. But the fact that you at least had the opportunity to talk about problems with your contact person was very important to me.* |
|  | Enabling factors for implementation | *I was looked after, treated and accompanied very individually and, above all, very flexibly adapted to my situation. So, everything was really, really great!*  *What I find really good is that there is not only care when you are an inpatient somewhere in the hospital, but also throughout the entire period of therapy.*  *I knew in the back of my mind that if something happened at home, I could call them. I think that's really good, to have someone you can call afterwards, because you know that if the world comes to an end, there's someone you can call. She'll tell you what to do, because she knows you.*  *In terms of organisation, I've always had the impression it's already an established programme.*  *So, the moment you actually got in touch and said, ‘I would like to take part in this programme’, that happened relatively quickly, very coordinated, very organised. Then you received the documents very quickly. It was processed relatively quickly and then you also got an appointment relatively quickly, and then it started quickly.* |
|  | Hindering factors for implementation | *I can imagine that many people are put off by this and say that they don't want to have anything to do with this kind of psycho stuff.*  *Nobody reads all those documents! I don't think anyone works through them. I didn't want to read it at all because I just didn't have the energy at that time. I wanted someone to take me by the hand, explain it to me and nothing more.*  *You have such a good programme, yes! But why doesn't everyone who deals with oncology patients know about it? I saw a flyer and wanted to get in. It wasn't so easy to find someone who knew about it.*  *I was told where I could turn to, including to these self-help groups. Then there are also individuals who can be called or with whom you can get in touch. So, I guess they are these kind of onco-guides.* |
| Optimisation needs | Meeting information needs | *It was actually until the end that I kind of didn't really know at all what each person in the programme was really doing.* |
|  | Picking up on information given by isPOonco-guides in the psychotherapeutical appointments | *It's such a double-edged sword. I mean, on the one hand you are looking for support so that you can cope with such things again. On the other hand, you only get the information, I'll say, addresses and where you can read up on it, but are you psychologically able to take it all in, […] in this situation? Perhaps the psycho-oncologist should also provide some other guidance, […] so that he or she can ask again and […]to take up the matter again or to support the person in some way […]* |
|  | Further group offers | *And what just occurred to me is that I would perhaps also like more connection between patients […], so that this barrier –is not so high? – that perhaps such courses would be offered again or that one at least says: […] that's where we can refer you and maybe with a bit of luck several cancer patients will actually register.* |
|  | Making the care period more flexible | *That may be the case for someone who, like me, finishes the treatment relatively quickly. But there are some who are in it for years. That's why you have to look at it individually. Some also come back because the cancer comes back again or some difficulties or questions arise much later.*  *I think it would be a good idea to ask about the need for follow-up care later on or to call again after two years. I think the normal cancer follow-up by the urologist also runs for five years. And maybe it would be a good idea for isPO to contact you again after one year and again until the end of these five years.* |
|  | More intensive psychosocial support | *So that's what I would be interested in, how to get a phone number or something. […] Of course, it would be a great thing to have someone who can advise you, because I could retire next year at 64. I have no idea about that.* |
|  | Selfhelp groups | *I also have to say that afterwards, when I was in the hospital with my operation and when I exchanged information with the other patient, I was somehow very grateful for the exchange and I can imagine that in particular, yes, that something like that would perhaps be interesting again, […] Maybe […] a kind of self-help group or something, right? So maybe something like that would be interesting again as a direct offer for someone.* |
|  | Support for relatives | *But I say, well, that would definitely be advisable, because the family suffers just as much as the person affected, right? I mean, it always affects a whole family […]it would be good if there was also support in this respect, right? So that they are advised or told "How do you deal with the disease?", that they can support them, […], because sometimes they are at a loss. I mean, my husband was also totally disoriented when he was confronted with it. That was a big shock for him* |
| isPO in routine care | Patient education | *For patients, I think it takes a lot of education, possibly even from the oncologist or the health insurance company, so that they know that there is such a programme and they are entitled to it and they don't need to be afraid to somehow tackle it. Maybe for some people this is a huge obstacle to say "Hey, I've just got something” and call the different therapists, no, tell them the whole thing first. So, you actually give it to the patient in small pieces and serve it to him on a silver platter.* |
|  | Interdisciplinary cooperation in oncology | *[…]it is best that this is also in one hand, i.e. that it is also recommended together with the doctors and that everything is in one hand, that there is really cooperation in this respect.* |
|  | Recognition at the political and social level | *and perhaps also be sensitised "Hey, people, the psyche, […] but at least just as important to get through at all is the psyche, […] and there, I think, are also many oncologists who very strongly underestimate that, so doctors in general[…]. I think that is still very important and for the health insurance companies, yes, that there is simply a huge need and I hope, yes, that through this feedback from us patients, their eyes will be opened that it is simply essential, important, because probably/ I don't know if you can prove it in any way, but that the boomerang will come back at some point.* |
|  | Wish for expansion | *Yes, that it will definitely be continued and further developed and that it will be offered at several locations, I would definitely recommend that. So, that many people can benefit from it.* |
